# Private sector tuberculosis prevention in the US: Characteristics associated with interferon-gamma release assay or tuberculin skin testing

**DOI:** 10.1371/journal.pone.0193432

**Published:** 2018-03-28

**Authors:** Erica L. Stockbridge, Thaddeus L. Miller, Erin K. Carlson, Christine Ho

**Affiliations:** 1 Department of Health Behavior and Health Systems, University of North Texas Health Science Center School of Public Health, Fort Worth, TX, United States of America; 2 Department of Advanced Health Analytics and Solutions, Magellan Health, Inc., Scottsdale, AZ, United States of America; 3 College of Nursing and Health Innovation, University of Texas at Arlington, Arlington, TX, United States of America; 4 Division of Tuberculosis Elimination, Centers for Disease Control and Prevention, Atlanta, GA, United States of America; Agencia de Salut Publica de Barcelona, SPAIN

## Abstract

**Objective:**

To determine whether latent tuberculosis infection risk factors are associated with an increased likelihood of latent tuberculosis infection testing in the US private healthcare sector.

**Data source:**

A national sample of medical and pharmacy claims representing services rendered January 2011 through December 2013 for 3,997,986 commercially insured individuals in the US who were 0 to 64 years of age.

**Study design:**

We used multivariable logistic regression models to determine whether TB/LTBI risk factors were associated with an increased likelihood of Interferon-Gamma Release Assay (IGRA) or Tuberculin Skin Test (TST) testing in the private sector.

**Principal findings:**

4.31% (4.27–4.34%) received at least one TST/IGRA test between 2011 and 2013 while 1.69% (1.67–1.72%) received a TST/IGRA test in 2013. Clinical risk factors associated with a significantly increased likelihood of testing included HIV, immunosuppressive therapy, exposure to tuberculosis, a history of tuberculosis, diabetes, tobacco use, end stage renal disease, and alcohol use disorder. Other significant variables included gender, age, asthma, the state tuberculosis rate, population density, and percent of foreign-born persons in a county.

**Conclusions:**

Private sector TST/IGRA testing is not uncommon and testing varies with clinical risk indicators. Thus, the private sector can be a powerful resource in the fight against tuberculosis. Analyses of administrative data can inform how best to leverage private sector healthcare toward tuberculosis prevention activities.

## Introduction

Tuberculosis (TB), an infectious disease caused by *Mycobacterium tuberculosis*, is one of the world’s deadliest diseases [1]. Although TB is less prevalent in the US than in many other countries, nearly 10,000 new TB cases are diagnosed in the US annually [2–4]. TB is a debilitating and potentially deadly illness with long-term health consequences and substantially increased mortality risk even after treatment is completed [5, 6]. Further, TB in the US exacts great financial and societal costs [5, 7, 8]. Consequently, domestic TB elimination, defined as a rate of less than one incident TB case per million population, has long been a goal of US public health policy [9–11].

It is generally accepted that this goal is achievable [3, 10, 12, 13], but the US falls well short of elimination, and advancement towards the goal has stalled [2, 4]. This is in part due to persistent heightened risk of active TB among the estimated 13 million people in the US with latent TB infection (LTBI) [9, 14, 15]. People with LTBI are infected with *M*. *tuberculosis* but do not have active TB disease. While they are asymptomatic and not infectious, on average 5 to 10% will progress to active TB in their lifetime if they are not treated [16]. Historically LTBI has been largely unaddressed, but well-targeted identification and treatment of people with LTBI have become important components of the domestic TB elimination strategy [11].

Public health authorities have led a coordinated effort against TB in the US, including providing much of the direct patient care associated with diagnosis and treatment of patients with active TB and LTBI [9, 17]. Private sector healthcare providers have played a less visible part of this work. However, recent recommendations from the US Preventive Services Task Force (USPSTF) and provisions of the Affordable Care Act create new incentives that may result in a growing presence of private sector healthcare in the domestic fight against TB [18–20]. Given the chronic constraints of public budgets, the potential to leverage private sector healthcare’s considerable resources toward an important public health goal is very attractive. Unfortunately, little information exists to guide policy makers as they consider the benefits and limitations of this new opportunity.

A key knowledge gap exists around risk-targeted LTBI testing and treatment in the private sector. LTBI is distributed heterogeneously within the US population. While roughly 5.0% of the US population has been estimated to have LTBI, prevalence is higher in some subpopulations (e.g., foreign-born persons) [14, 15]. Similarly, the risk of progression to active TB among those with LTBI varies, with certain characteristics increasing the risk of progression (e.g., immunosuppression, diabetes) [16, 19, 21]. Conversely, many people are at little risk of *M*. *tuberculosis* infection or disease progression. When low-risk people are tested, the harms may outweigh the benefits [18]. There is a high probability of false-positive results [22], and the commonly used treatment regimen with isoniazid is long and carries a not insignificant risk of hepatotoxicity and other side effects [23, 24]. Thus, LTBI testing should be targeted toward individuals and populations with known risks [25]. While it is known that some testing already occurs in the US private sector [26], it is unknown whether such testing is well-targeted. Understanding the appropriateness of LTBI testing occurring within this increasingly important setting is necessary in order for public health leaders to shape the delivery of these services in the future. We analyzed a large commercial claims dataset to determine whether TB/LTBI risk factors are associated with an increased likelihood of TST or IGRA testing.

## Methods

This research was approved by the University of North Texas Health Science Center institutional review board as exempt category research.

### Data source

We used the Optum Clinformatics Data Mart Database to examine pharmacy and medical insurance claims for a randomly selected, de-identified sample of 4 million people ages 0 to 64 years who had continuous commercial insurance coverage between January 1, 2011 and December 31, 2013 [27]. Approximately 19% of the commercially insured US population is represented in this database. The data included information about each individual’s insurance-covered prescriptions filled and healthcare services received during that three-year period, at minimum. The sample roughly approximated the 2010 US population geographic distribution by Census division [28]. Individuals with missing geographic information (i.e., census division, rural-urban continuum category, state TB rate, or county characteristics) were excluded from analysis (n = 2,014; 0.05%).

### Measures

#### Outcome variable

The outcome of interest was the receipt of at least one tuberculin skin test (TST) or interferon-gamma release assay (IGRA). We used current procedural terminology (CPT) codes to identify testing by TST or either of the IGRA methods (i.e., T-SPOT^®^.TB or QuantiFERON^®^–TB). In addition, we presumed that ICD-9-CM coding indicating “special screening examination for pulmonary tuberculosis, including diagnostic skin testing” represented a TST when not accompanied by a testing CPT code. They were counted as testing occurrences if they existed in the absence of a CPT code for a TST, IGRA, or another procedure potentially related to *M*. *tuberculosis* testing within ±3 days from the date of service (see S1 File). Presumptive TST screenings were not combined with CPT-coded TST screenings when testing methods were analyzed separately, but when testing methods were examined in total they were included in the total. Testing with TSTs or IGRAs is henceforth collectively referred to as “TST/IGRA testing.”

#### Explanatory variables

We constructed explanatory variables from information in the medical and pharmacy claims data based on services occurring and prescriptions filled between 2011 and 2013. Socio-demographic variables included sex, age, census region, and urban-rural classification [29]. Additional variables included insurance type (health maintenance organization [HMO], indemnity, point of service [POS], or preferred provider organization [PPO]) and residence in a county designated as a geographic primary care physician health professional shortage area (PCP-HPSA) [30]. We incorporated indicators of asthma and chronic obstructive pulmonary disease (COPD) as well as variables associated with risk of LTBI or progression to active TB, including the state TB rate [31]. The percentage of households living under the federal poverty level (FPL) in an individual’s county was included as a proxy for household income [32]. Country of birth was unknown, but the prevalence of foreign-born individuals in the county served as a rough measure of nativity [32]. Clinical risk factors were incorporated, including HIV, use of immunosuppressive medication, contact with or exposure to TB, a history of TB, diabetes, evidence of tobacco use, leukemia or lymphoma, lung cancer, head or neck cancer, lung disease due to external agents (e.g., silicosis), gastrectomy or gastric bypass, end stage renal disease/dialysis, alcohol use disorder, and drug use disorder [25]. See Supplemental File 2 (S2 File) for additional details. We used a simple count of each individual’s clinical risk factors to assign cumulative risk (i.e., 6 levels of risk representing 0 risk factors through ≥5 risk factors).

### Statistical analyses

We calculated the proportion of individuals receiving at least one test, examining each type of test separately (i.e., TST, presumptive TST, IGRA QFT, IGRA T-spot, IGRA in total) and with all testing methods combined. We estimated these proportions for two time periods: 1) January 2011 through December 2013 (the longest period for which complete data were available) and 2) January through December 2013 (a subperiod representing the most recent calendar year). The unit of measure for these proportions was individual people; those receiving >1 test in a given time period were only counted one time in the numerator. Examining the likelihood of testing both in a single year and in a longer observation period is useful because it illustrates how a single year of data provides an incomplete picture of LTBI testing within a group of people.

Additionally, testing rates were calculated based on a count of the total number of tests between 2011 and 2013 divided by person-years. As all individuals had continuous insurance coverage for three years, we calculated person-years by multiplying the number of individuals included in the analysis by three. All testing occurrences were represented in these testing rates; when an individual had multiple tests all of these tests were counted.

We examined the bivariate relationships between explanatory variables and TST/IGRA testing (combined) between 2011 and 2013 using chi square tests for categorical variables and Spearman correlations for continuous variables. We then explored adjusted associations between these variables and TST/IGRA testing with two logistic regression models. Model 1 includes the specific clinical risk factors as explanatory variables while Model 2 includes a count of clinical risk factors. To provide insight into effect sizes and practical significance of the observed statistically significant differences, the models were used to generate the average adjusted probability of a TST/IGRA for each level of the categorical explanatory variables and for the minimum, 25^th^ percentile, median, 75^th^ percentile, and maximum values of the continuous explanatory variables. These probabilities were expressed as percentages, and they represent the average predicted probability of a TST/IGRA conditional on all observations having the given value. All statistical testing used Stata 14.2 [StataCorp, College Station, TX] and was two-sided. Given the large sample size and multiple comparisons [33], significance was tested at p < 0.001.

## Results

Of 3,997,986 people with sufficiently detailed geographic data for inclusion in analyses, 172,253 (4.3%) received ≥1 TST/IGRA test between 2011 and 2013 and 67,792 (1.7%) received ≥1 TST/IGRA test in 2013 (Table 1). The TST/IGRA testing rate was 1902.9/100,000 person-years. TSTs were more prevalent than IGRAs in both periods studied. Between 2011 and 2013, 3.8% of individuals received ≥1 TST but 0.4% received ≥1 IGRA. In 2013, 1.4% received ≥1 TST while 0.2% received ≥1 IGRA. Presumptive TST screening (inferred from ICD-9-CM codes but not coded with a CPT code) was identified at a rate of 96.6/100,000 person-years, representing 11,584 (5.1%; 99.9% Confidence Interval: 4.9, 5.2) of the 228,230 tests conducted.

**Table 1:** Rates of screening for *Mycobacterium tuberculosis* with tuberculin skin tests (TSTs) or interferon-gamma release assays (IGRAs) in commercially insured individuals ages 0 to 64 years, based on data from the Optum Clinformatics Data Mart Database (N = 3,997,986).

Method	# Tests, 2011–2013	Tests per 100,000 Person-Years, 011–2013	% of Insured Persons with ≥ 1 Test, 2011–2013 (99.9% Confidence Interval)	% of Insured Persons with ≥ 1 Test, 2013 (99.9% Confidence Interval)
Tuberculin skin test (TST)	197,980	1650.66	3.83% (3.80–3.86%)	1.42% (1.40–1.44%)
Interferon-gamma release assay (IGRA)*	18,666	155.63	0.39% (0.38–0.40%)	0.22% (0.21–0.22%)
QuantiFERON	17,644	147.11	0.37% (0.36–0.38%)	0.20% (0.20-.021%)
T-SPOT	1,022	8.52	0.02% (0.02–0.03%)	0.01% (0.01–0.01%)
TST screening likely occurred but procedure code not specified**	11,584	96.58	0.21% (0.21–0.22%)	0.09% (0.08–0.09%)
Total (All combined)*	228,230	1902.87	4.31% (4.27–4.34%)	1.69% (1.67–1.72%)

* Percentage totals may be different from the sum of the individual screening percentages for two reasons: 1) Rounding, and 2) Some individuals were screened >1 time in a given time period.

** Based on the presence of the ICD-9-CM diagnosis code “V74.1: Special screening examination for pulmonary tuberculosis, including diagnostic skin testing” on a given date, excluding those with a current procedural terminology (CPT) procedure code for a TST, IGRA, or other procedure potentially related to *M*. *tuberculosis* screening occurring within ±3 days from that date.

Most observable clinical risk factors were independently and cumulatively associated with an increased likelihood of TST/IGRA testing (Tables 2 and 3). These included HIV, use of immunosuppressive medications, contact with or exposure to TB, a history of TB, diabetes, tobacco use, lung disease due to external agents, end stage renal disease/dialysis, and alcohol use disorder. Asthma, while not a clinical risk factor for LTBI or progression, was also significantly associated with TST/IGRA testing.

**Table 2:** Frequency distributions of variables describing commercially insured individuals ages 0 to 64 years and the proportion of people with these characteristics who were screened for *Mycobacterium tuberculosis* with a tuberculin skin test (TST) or an interferon-gamma release assay (IGRA) between 2011 and 2013, based on data from the Optum Clinformatics Data Mart Database (N = 3,997,986). All numbers and percentages in this table are unadjusted.

		N	% or Mean of Total	No Screening (% or Mean)	Had Screening (% or Mean)	*P*-value
**Sex**	Female	2,021,984	50.6%	94.9%	5.1%	<.001
	Male	1,976,002	49.4%	96.5%	3.5%	
**Age**	0–4	192,115	4.8%	91.4%	8.6%	<.001
	5–9	284,868	7.1%	95.1%	4.9%	
	10–14	313,776	7.9%	95.0%	5.0%	
	15–19	325,691	8.2%	88.4%	11.6%	
	20–24	246,268	6.2%	91.9%	8.1%	
	25–29	207,736	5.2%	96.9%	3.1%	
	30–34	286,912	7.2%	96.8%	3.2%	
	35–39	320,717	8.0%	96.9%	3.1%	
	40–44	384,974	9.6%	97.2%	2.8%	
	45–49	416,863	10.4%	97.6%	2.4%	
	50–54	432,965	10.8%	97.8%	2.2%	
	55–59	390,435	9.8%	97.9%	2.1%	
	60–64	194,666	4.9%	98.0%	2.0%	
**Census Division**	New England	412,136	10.3%	96.6%	3.4%	<.001
	Mid-Atlantic	660,516	16.5%	91.8%	8.2%	
	East North Central	660,596	16.5%	96.6%	3.4%	
	West North Central	373,219	9.3%	97.2%	2.8%	
	South Atlantic	568,544	14.2%	96.3%	3.8%	
	East South Central	137,765	3.5%	97.5%	2.6%	
	West South Central	694,018	17.4%	97.5%	2.6%	
	Mountain	198,636	5.0%	97.2%	2.8%	
	Pacific	292,556	7.3%	92.1%	7.9%	
**Rural-Urban Continuum Category**	Large central metro (Most urban & densely populated)	1,114,746	27.9%	94.5%	5.5%	<.001
	Large fringe metro	1,518,188	38.0%	95.3%	4.7%	
	Medium metro	763,457	19.1%	96.6%	3.4%	
	Small metro	269,069	6.7%	97.5%	2.5%	
	Micropolitan	201,185	5.0%	97.7%	2.3%	
	Noncore (Most rural & least populated)	131,341	3.3%	97.9%	2.1%	
**PCP Health Professional Shortage Area**	Not an HPSA	3,854,171	96.4%	95.6%	4.4%	<.001
	HPSA	143,815	3.6%	97.9%	2.1%	
**Insurance Type**	HMO	635,718	15.9%	95.1%	4.9%	<.001
	Indemnity	1,585	0.0%	97.7%	2.3%	
	POS	2,712,259	67.8%	95.9%	4.1%	
	PPO	648,424	16.2%	95.3%	4.7%	
Percent of Households in County with Income under FPL*		3,997,986	14.44(0.003)	14.46(0.003)	13.95(0.014)	<.001
	0 to 10%	986,695	24.7%	94.9%	5.1%	<.001
	>10 to 15%	1,222,283	30.6%	95.8%	4.2%	
	>15 to 20%	1,259,323	31.5%	96.0%	4.0%	
	>20%	529,685	13.2%	96.0%	4.0%	
Percent of Foreign-born Individuals in County*		3,997,986	12.77(0.005)	12.56(0.005)	17.57(0.028)	<.001
	0 to 5%	1,098,591	27.5%	97.4%	2.6%	<.001
	>5 to 10%	956,521	23.9%	96.6%	3.4%	
	>10 to 20%	1,051,342	26.3%	96.2%	3.8%	
	>20%	891,532	22.3%	92.0%	8.0%	
State TB Rate per 100,000*		3,997,986	3.11(0.001)	3.09(0.001)	3.57(0.003)	<.001
	0 to 1.5%	842,317	21.1%	97.2%	2.8%	<.001
	>1.5 to 3%	845,383	21.1%	96.5%	3.5%	
	>3 to 4%	1,275,186	31.9%	96.3%	3.7%	
	>4%	1,035,100	25.9%	93.0%	7.0%	
**Asthma**	No diagnosis	3,768,168	94.3%	95.8%	4.2%	<.001
	Had diagnosis	229,818	5.8%	93.8%	6.2%	
**COPD**	No diagnosis	3,956,823	99.0%	95.7%	4.3%	.001
	Had diagnosis	41,163	1.0%	95.4%	4.6%	
**Count of Clinical Risk Factors**	0 clinical risk factors	3,523,528	88.1%	96.0%	4.0%	<.001
	1 clinical risk factor	407,710	10.2%	94.0%	6.0%	
	2 clinical risk factors	57,922	1.5%	91.5%	8.5%	
	3 clinical risk factors	9,729	0.2%	88.9%	11.1%	
	4 clinical risk factors	998	0.0%	85.0%	15.1%	
	> = 5 clinical risk factors	113	0.0%	76.2%	23.8%	
**HIV**	No diagnosis	3,989,327	99.8%	95.7%	4.3%	<.001
	Had diagnosis	8,659	0.2%	76.8%	23.3%	
**Immuno-supressive Medications**	No medication/procedure	3,955,046	98.9%	96.0%	4.1%	<.001
	Had medication/procedure	42,940	1.1%	71.8%	28.2%	
**Diagnosis of Contact with TB**	No diagnosis	3,990,955	99.8%	95.8%	4.2%	<.001
	Had diagnosis	7,031	0.2%	16.3%	83.7%	
**History/Late Effects of TB**	No diagnosis	3,997,034	100.0%	95.7%	4.3%	<.001
	Had diagnosis	952	0.0%	79.8%	20.2%	
**Diabetes**	No diagnosis/medication	3,764,124	94.2%	95.6%	4.4%	<.001
	Had diagnosis/medication	233,862	5.9%	96.9%	3.1%	
**Tobacco**	No diagnosis/medication	3,825,292	95.7%	95.7%	4.4%	<.001
	Had diagnosis/medication	172,694	4.3%	96.6%	3.4%	
**Leukemia or Lymphoma**	No diagnosis	3,986,952	99.7%	95.7%	4.3%	<.001
	Had diagnosis	11,034	0.3%	95.0%	5.0%	
**Lung Cancer**	No diagnosis	3,994,927	99.9%	95.7%	4.3%	.148
	Had diagnosis	3,059	0.1%	95.2%	4.8%	
**Head or Neck Cancer**	No diagnosis	3,994,510	99.9%	95.7%	4.3%	<.001
	Had diagnosis	3,476	0.1%	97.0%	3.0%	
**Lung Disease Due to External Agents**	No diagnosis	3,997,416	100.0%	95.7%	4.3%	<.001
	Had diagnosis	570	0.0%	91.4%	8.6%	
**Gastrectomy or Gastric Bypass**	No diagnosis/procedure	3,978,496	99.5%	95.7%	4.3%	.351
	Had diagnosis/procedure	19,490	0.5%	95.8%	4.2%	
**ESRD/Dialysis**	No diagnosis	3,991,465	99.8%	95.7%	4.3%	<.001
	Had diagnosis	6,521	0.2%	84.5%	15.6%	
**Alcohol Use Disorder**	No diagnosis	3,964,501	99.2%	95.7%	4.3%	<.001
	Had diagnosis	33,485	0.8%	94.7%	5.3%	
**Drug Use Disorder**	No diagnosis	3,965,294	99.2%	95.7%	4.3%	<.001
	Had diagnosis	32,692	0.8%	93.5%	6.5%	

Totals may sum to >100% due to rounding.

* The percent of households in county with income under FPL, the percent of foreign-born individuals in county, and the state TB rate were entered into the models as continuous variables. The values in these rows represent the total N, the overall mean and standard error, and the mean and standard error for tested and untested individuals, respectively. The rows following the headers (e.g., 0 to 10%, >10 to 15%, etc.) represent categorizations of the continuous variables, and the number of individuals with values falling into each category are counted in the row.

Abbreviations:

TST = tuberculin skin test

IGRA = interferon-gamma release assay

PCP = primary care provider

TB = tuberculosis

FPL = federal poverty level

COPD = chronic obstructive pulmonary disease

HIV = human immunodeficiency virus

ESRD = end stage renal disease

HMO = health maintenance organization

PPO = preferred provider organization

POS = place of service

**Table 3:** Results of two logistic regression models which examine associations between insurance enrollee characteristics and screening for *Mycobacterium tuberculosis* with either a tuberculin skin test (TST) or an interferon-gamma release assay (IGRA) between 2011 and 2013, based on data from the Optum Clinformatics Data Mart Database (N = 3,997,986).

		Model #1: Includes Individual Clinical Risk Factors							Model #2: Includes a Count of Clinical Risk Factors						
		Odds Ratio	*P*-value	99.9% Confidence Interval		Average Adjusted Prob-ability*	99.9% Confidence Interval		Odds Ratio	*P*-value	99.9% Confidence Interval		Average Adjusted Prob-ability*	99.9% Confidence Interval	
**Sex**	Female	1.00				5.1%	5.1%	5.2%	1.00				5.2%	5.2%	5.3%
	Male	0.65	<.001	0.63	0.66	3.5%	3.5%	3.5%	0.63	<.001	0.62	0.64	3.4%	3.4%	3.5%
**Age**	0–4	1.00				9.0%	8.8%	9.2%	1.00				9.7%	9.5%	10.0%
	5–9	0.55	<.001	0.53	0.57	5.4%	5.3%	5.6%	0.55	<.001	0.53	0.57	5.8%	5.6%	5.9%
	10–14	0.57	<.001	0.55	0.59	5.6%	5.5%	5.7%	0.57	<.001	0.55	0.59	5.9%	5.8%	6.1%
	15–19	1.47	<.001	1.42	1.52	12.4%	12.2%	12.5%	1.41	<.001	1.36	1.46	12.9%	12.7%	13.1%
	20–24	0.93	<.001	0.89	0.96	8.5%	8.3%	8.6%	0.87	<.001	0.84	0.90	8.7%	8.5%	8.8%
	25–29	0.30	<.001	0.28	0.31	3.2%	3.0%	3.3%	0.29	<.001	0.28	0.31	3.2%	3.1%	3.3%
	30–34	0.30	<.001	0.29	0.31	3.2%	3.1%	3.3%	0.29	<.001	0.28	0.31	3.2%	3.1%	3.3%
	35–39	0.28	<.001	0.26	0.29	3.0%	2.9%	3.1%	0.27	<.001	0.26	0.28	3.0%	2.9%	3.1%
	40–44	0.25	<.001	0.24	0.26	2.7%	2.6%	2.8%	0.24	<.001	0.23	0.25	2.7%	2.6%	2.8%
	45–49	0.21	<.001	0.20	0.22	2.3%	2.2%	2.4%	0.20	<.001	0.20	0.21	2.3%	2.2%	2.4%
	50–54	0.19	<.001	0.18	0.20	2.1%	2.0%	2.2%	0.18	<.001	0.17	0.19	2.0%	1.9%	2.1%
	55–59	0.18	<.001	0.17	0.19	2.0%	1.9%	2.1%	0.17	<.001	0.16	0.18	1.9%	1.8%	1.9%
	60–64	0.16	<.001	0.15	0.17	1.8%	1.7%	1.9%	0.15	<.001	0.14	0.16	1.7%	1.6%	1.7%
**Census Division**	New England	1.00				3.5%	3.4%	3.6%	1.00				3.4%	3.3%	3.5%
	Mid-Atlantic	1.96	<.001	1.89	2.03	6.3%	6.2%	6.4%	1.98	<.001	1.91	2.05	6.4%	6.3%	6.5%
	East North Central	1.51	<.001	1.44	1.58	5.0%	4.9%	5.1%	1.50	<.001	1.44	1.57	5.0%	4.8%	5.1%
	West North Central	1.19	<.001	1.13	1.25	4.1%	3.9%	4.2%	1.19	<.001	1.13	1.24	4.0%	3.9%	4.1%
	South Atlantic	1.06	<.001	1.02	1.10	3.7%	3.6%	3.7%	1.08	<.001	1.03	1.12	3.7%	3.6%	3.7%
	East South Central	1.13	<.001	1.06	1.21	3.9%	3.7%	4.1%	1.14	<.001	1.06	1.22	3.8%	3.6%	4.1%
	West South Central	0.70	<.001	0.67	0.74	2.5%	2.5%	2.6%	0.72	<.001	0.69	0.75	2.5%	2.4%	2.6%
	Mountain	1.06	.001	1.00	1.13	3.7%	3.5%	3.8%	1.08	<.001	1.02	1.15	3.7%	3.5%	3.8%
	Pacific	1.47	<.001	1.40	1.55	4.9%	4.8%	5.0%	1.48	<.001	1.41	1.55	4.9%	4.7%	5.0%
**Rural-Urban Continuum Category**	Large central metro (Most urban & densely populated)	1.00				4.3%	4.2%	4.4%	1.00				4.3%	4.3%	4.4%
	Large fringe metro	1.05	<.001	1.02	1.08	4.5%	4.4%	4.6%	1.04	<.001	1.02	1.07	4.5%	4.4%	4.6%
	Medium metro	1.00	.964	0.97	1.03	4.3%	4.2%	4.4%	0.98	.022	0.95	1.01	4.3%	4.2%	4.3%
	Small metro	0.86	<.001	0.82	0.90	3.8%	3.6%	3.9%	0.84	<.001	0.80	0.88	3.7%	3.6%	3.9%
	Micropolitan	0.80	<.001	0.76	0.85	3.5%	3.4%	3.7%	0.78	<.001	0.74	0.82	3.5%	3.3%	3.6%
	Noncore (Most rural & least populated)	0.81	<.001	0.75	0.87	3.6%	3.3%	3.8%	0.79	<.001	0.73	0.84	3.5%	3.3%	3.7%
**PCP Health Professional Shortage Area**	Not an HPSA	1.00				4.3%	4.3%	4.3%	1.00				4.3%	4.3%	4.4%
	HPSA	0.93	<.001	0.87	0.99	4.0%	3.8%	4.3%	0.92	<.001	0.86	0.98	4.0%	3.8%	4.2%
**Insurance Type**	HMO	1.00				4.2%	4.1%	4.3%	1.00				4.2%	4.1%	4.3%
	Indemnity	0.76	.110	0.43	1.34	3.3%	1.6%	4.9%	0.73	.068	0.42	1.29	3.1%	1.5%	4.8%
	POS	1.03	<.001	1.00	1.06	4.3%	4.3%	4.3%	1.02	.005	1.00	1.05	4.3%	4.2%	4.3%
	PPO	1.09	<.001	1.06	1.12	4.5%	4.4%	4.6%	1.10	<.001	1.07	1.13	4.6%	4.5%	4.6%
Percent of Households in County with Income under FPL***	1.00	.004	1.00	1.00	**	**	**	1.00	.001	1.00	1.00	**	**	**
Percent of Foreign-born Individuals in County***	1.02	<.001	1.02	1.02	**	**	**	1.02	<.001	1.02	1.02	**	**	**
State TB Rate per 100,000****	1.18	<.001	1.17	1.19	**	**	**	1.18	<.001	1.16	1.19	**	**	**
**Asthma**	No diagnosis	1.00				4.2%	4.2%	4.3%	1.00				4.2%	4.2%	4.3%
	Had diagnosis	1.31	<.001	1.26	1.35	5.3%	5.2%	5.5%	1.27	<.001	1.23	1.31	5.3%	5.1%	5.4%
**COPD**	No diagnosis	1.00				4.3%	4.3%	4.3%	1.00				4.3%	4.3%	4.3%
	Had diagnosis	1.48	<.001	1.36	1.62	6.0%	5.6%	6.5%	1.16	<.001	1.06	1.25	4.9%	4.5%	5.3%
**Count of Clinical Risk Factors**	0 clinical risk factors	N/A				N/A	N/A	N/A	1.000				3.8%	3.8%	3.8%
	1 clinical risk factor	N/A	N/A	N/A	N/A	N/A	N/A	N/A	2.82	<.001	2.75	2.89	9.4%	9.2%	9.6%
	2 clinical risk factors	N/A	N/A	N/A	N/A	N/A	N/A	N/A	4.23	<.001	4.01	4.45	13.0%	12.5%	13.5%
	3 clinical risk factors	N/A	N/A	N/A	N/A	N/A	N/A	N/A	4.91	<.001	4.41	5.47	14.6%	13.4%	15.8%
	4 clinical risk factors	N/A	N/A	N/A	N/A	N/A	N/A	N/A	9.70	<.001	7.35	12.80	23.6%	19.3%	27.8%
	> = 5 clinical risk factors	N/A	N/A	N/A	N/A	N/A	N/A	N/A	18.51	<.001	9.39	36.49	34.8%	21.8%	47.7%
**HIV**	No diagnosis	1.00				4.3%	4.2%	4.3%	N/A				N/A	N/A	N/A
	Had diagnosis	11.02	<.001	10.05	12.08	26.7%	25.2%	28.2%	N/A	N/A	N/A	N/A	N/A	N/A	N/A
**Immuno-supressive Medications**	No medication/ procedure	1.00				4.0%	4.0%	4.1%	N/A				N/A	N/A	N/A
	Had medication/ procedure	17.51	<.001	16.79	18.26	34.4%	33.7%	35.2%	N/A	N/A	N/A	N/A	N/A	N/A	N/A
**Diagnosis of Contact with TB**	No diagnosis	1.00				4.2%	4.2%	4.2%	N/A				N/A	N/A	N/A
	Had diagnosis	108.88	<.001	97.05	122.14	71.6%	69.6%	73.7%	N/A	N/A	N/A	N/A	N/A	N/A	N/A
**History/Late Effects of TB**	No diagnosis	1.00				4.3%	4.3%	4.3%	N/A				N/A	N/A	N/A
	Had diagnosis	4.47	<.001	3.22	6.19	14.5%	11.0%	18.0%	N/A	N/A	N/A	N/A	N/A	N/A	N/A
**Diabetes**	No diagnosis/ medication	1.00				4.3%	4.3%	4.3%	N/A				N/A	N/A	N/A
	Had diagnosis/ medication	1.15	<.001	1.10	1.20	4.8%	4.7%	5.0%	N/A	N/A	N/A	N/A	N/A	N/A	N/A
**Tobacco**	No diagnosis/ medication	1.00				4.3%	4.3%	4.3%	N/A				N/A	N/A	N/A
	Had diagnosis/ medication	1.12	<.001	1.07	1.18	4.7%	4.5%	4.9%	N/A	N/A	N/A	N/A	N/A	N/A	N/A
**Leukemia or Lymphoma**	No diagnosis	1.00				4.3%	4.3%	4.4%	N/A				N/A	N/A	N/A
	Had diagnosis	0.55	<.001	0.46	0.64	2.5%	2.2%	2.9%	N/A	N/A	N/A	N/A	N/A	N/A	N/A
**Lung Cancer**	No diagnosis	1.00				4.3%	4.3%	4.3%	N/A				N/A	N/A	N/A
	Had diagnosis	1.35	.001	0.99	1.84	5.6%	4.1%	7.0%	N/A	N/A	N/A	N/A	N/A	N/A	N/A
**Head or Neck Cancer**	No diagnosis	1.00				4.3%	4.3%	4.3%	N/A				N/A	N/A	N/A
	Had diagnosis	1.01	.923	0.71	1.44	4.4%	3.0%	5.7%	N/A	N/A	N/A	N/A	N/A	N/A	N/A
**Lung Disease Due to External Agents**	No diagnosis	1.00				4.3%	4.3%	4.3%	N/A				N/A	N/A	N/A
	Had diagnosis	1 800	<.001	1.04	3.13	7.1%	3.8%	10.4%	N/A	N/A	N/A	N/A	N/A	N/A	N/A
**Gastrectomy or Gastric Bypass**	No diagnosis/ procedure	1.00				4.3%	4.3%	4.3%	N/A				N/A	N/A	N/A
	Had diagnosis/ procedure	1.31	<.001	1.16	1.49	5.4%	4.9%	6.0%	N/A	N/A	N/A	N/A	N/A	N/A	N/A
**ESRD/Dialysis**	No diagnosis	1.00				4.3%	4.3%	4.3%	N/A				N/A	N/A	N/A
	Had diagnosis	1.40	<.001	1.22	1.60	5.7%	5.1%	6.4%	N/A	N/A	N/A	N/A	N/A	N/A	N/A
**Alcohol Use Disorder**	No diagnosis	1.00				4.3%	4.3%	4.3%	N/A				N/A	N/A	N/A
	Had diagnosis	1.29	<.001	1.17	1.41	5.3%	4.9%	5.8%	N/A	N/A	N/A	N/A	N/A	N/A	N/A
**Drug Use Disorder**	No diagnosis	1.00				4.3%	4.3%	4.3%	N/A				N/A	N/A	N/A
	Had diagnosis	1.06	.020	0.98	1.16	4.5%	4.2%	4.9%	N/A	N/A	N/A	N/A	N/A	N/A	N/A

* Calculated as the average predicted probability of a test conditional on all observations being in the category represented by the row. The difference between the predicted probabilities for two categories of a given categorical variable represents the average marginal effect.

** The percent of households in county with income under FPL, percent of foreign-born individuals in county, and the state TB rate were entered into the models as continuous variables. Average predicted probability of *Mycobacterium tuberculosis* screening at the minimum, maximum, and quartile values of these variables can be found in Table 4.

*** Unit of increase is 1 per cent.

**** Unit of increase is 1 per 100,000.

Abbreviations:

TST = tuberculin skin test

IGRA = interferon-gamma release assay

PCP = primary care provider

TB = tuberculosis

FPL = federal poverty level

COPD = chronic obstructive pulmonary disease

HIV = human immunodeficiency virus

ESRD = end stage renal disease

HMO = health maintenance organization

PPO = preferred provider organization

POS = place of service

**Table 4:** Average adjusted probability of *Mycobacterium tuberculosis* screening with either a tuberculin skin test (TST) or an interferon-gamma release assay (IGRA) at the minimum, maximum, and quartile values of the continuous variables included in the two logistic regression models detailed in Table 3. These models examine associations between insurance enrollee characteristics and screenings for *M*. *tuberculosis* between 2011 and 2013, based on data from the Optum Clinformatics Data Mart Database (N = 3,997,986).

		Percent of Foreign-born Individuals in County *Models 1 & 2 P<0*.*001*				Percent of Households in County with Income under FPL *Model 1 P = 0*.*004*, *Model 2 P = 0*.*001*				State TB Rate per 100,000 *Models 1 & 2 P<0*.*001*			
		% Foreign-born	Average Adjusted Prob-ability*	99.9% Confidence Interval		% Under FPL	Average Adjusted Prob-ability*	99.9% Confidence Interval		State TB Rate	Average Adjusted Prob-ability*	99.9% Confidence Interval	
**Model #1: Includes Individual Clinical Risk Factors**	Minimum	0.0%	3.2%	3.2%	3.3%	3.1%	4.4%	4.3%	4.5%	0.4	2.8%	2.7%	2.9%
	25^th^ percentile	4.8%	3.5%	3.5%	3.6%	10.2%	4.3%	4.3%	4.4%	1.8	3.4%	3.4%	3.5%
	Median	9.3%	3.8%	3.8%	3.9%	13.9%	4.3%	4.3%	4.3%	3.2	4.2%	4.2%	4.2%
	75^th^ percentile	19.0%	4.6%	4.5%	4.6%	18.2%	4.3%	4.2%	4.3%	4.4	5.0%	4.9%	5.0%
	Maximum	51.2%	8.1%	7.8%	8.4%	51.2%	4.1%	3.8%	4.3%	9.0	9.4%	8.9%	9.9%
**Model #2: Includes a Count of Clinical Risk Factors**	Minimum	0.0%	3.1%	3.1%	3.2%	3.1%	4.4%	4.3%	4.5%	0.4	2.7%	2.7%	2.8%
	25^th^ percentile	4.8%	3.4%	3.4%	3.5%	10.2%	4.3%	4.3%	4.4%	1.8	3.4%	3.3%	3.5%
	Median	9.3%	3.8%	3.7%	3.8%	13.9%	4.3%	4.3%	4.3%	3.2	4.2%	4.2%	4.2%
	75^th^ percentile	19.0%	4.6%	4.5%	4.6%	18.2%	4.3%	4.2%	4.3%	4.4	5.0%	4.9%	5.1%
	Maximum	51.2%	8.6%	8.2%	8.9%	51.2%	4.0%	3.7%	4.3%	9.0	9.5%	9.0%	10.1%

* Calculated as the average predicted probability of a test conditional on all observations being at the value represented by the row.

Abbreviations:

TST = Tuberculin skin test

IGRA = interferon-gamma release assay

FPL = federal poverty level

Most non-clinical explanatory variables were also significantly associated with TST/IGRA testing in adjusted and unadjusted models (Table 4). Females were more likely to be tested than males. There was higher likelihood of testing among very young children and young adults with a decreasing trend as age increased beyond 24 years. Testing likelihood rose with the state TB rate, with increased population density, with larger relative populations of foreign-born persons in a county, and with less restrictive insurance. Living in a PCP-HPSA was associated with decreased testing likelihood.

Having COPD or a gastrectomy/gastric bypass was not significantly associated with testing in the bivariate analyses but were associated with a higher likelihood of testing in the multivariable models. Conversely, having a drug use disorder was significantly associated with TST/IGRA testing in bivariate analyses but was non-significant in the multivariable model. Having leukemia or lymphoma was associated with an increased likelihood of testing in the unadjusted analysis but a decreased likelihood in the adjusted analysis. Head/neck cancer was associated with a lower likelihood of testing in the unadjusted analysis but was non-significant in the adjusted analysis. Lung cancer was not significantly associated with testing in either the unadjusted or adjusted analyses.

## Discussion

Our study provides evidence that the US private healthcare sector has actively participated in domestic TB prevention-related activities in recent years, even prior to the USPSTF recommendations. Our results provide an important window into the relative likelihood of LTBI testing for given patient groups to identify those more or less likely to be tested by broadly observable characteristics. More than 1 in 25 (4.31%) commercially insured individuals in our sample received either a TST or IGRA during three years of observation, and likelihood of screening closely tracked important clinical and other risk factors.

The majority of the characteristics known to be associated with increased risk of *M*. *tuberculosis* infection or disease progression were found to be associated with an increased likelihood of TST/IGRA testing, and groups at the highest risk (contact with or exposure to TB, immunosuppressive therapy, and HIV) had the highest adjusted probability of testing (71.6%, 34.4%, and 26.7%, respectively). These findings are heartening, as they suggest that guideline-concordant testing is occurring in the private sector. Testing is strongly recommended for persons in these groups, since they are at the highest risk for developing active TB if they are infected with *M*. *tuberculosis* [25]. We also found that as the number of clinical risk factors for a given person increased so did the likelihood that he or she would be tested. In combination, these results suggest that many private sector providers are aware of the factors most strongly associated with TB and they conduct TB/LTBI testing accordingly.

The clinical characteristics associated with an intermediate risk of *M*. *tuberculosis* infection or disease progression (i.e., those at increased risk but not the highest risk as defined above) were also generally associated with a statistically significant increase in the likelihood of testing. However, the magnitude of the effects were not as striking as those seen when examining high-risk characteristics. For example, the average adjusted probability of testing for someone with end stage renal disease was 5.7% versus 4.3% for someone without, and the average adjusted probability of testing for someone with diabetes was 4.8% versus 4.3% for someone without. Additionally, some clinical risk factors were not associated with an increased likelihood of testing (e.g., head/neck cancer, lung cancer). These mixed results align with the current lack of clear guidance regarding LTBI testing for US patients with intermediate-risk conditions.

US Centers for Disease Control and Prevention (CDC) guidelines indicate that diabetes, chronic renal failure, alcohol abuse, and a number of other clinical conditions increase the risk of developing TB. That said, increased risk may not warrant targeted LTBI screening of individuals with these conditions. Recent screening guidelines released by the World Health Organization (WHO) recommend that individuals with diabetes, alcohol use disorders, and certain other intermediate risk conditions not be tested for LTBI unless they have other risk factors specified in the guidelines [34]. To date, national public health organizations have not released detailed clinical practice guidelines specifying which patient groups in the US are and are not appropriate for LTBI screening [21, 25] although such guidelines are in development by the American Thoracic Society (ATS) / Infectious Disease Society of America (IDSA) / CDC [13]. These guidelines will be an important step forward and are expected to be well-received by private sector providers; the USPSTF received much feedback on their draft LTBI screening recommendation requesting that they clarify which patient populations should be tested [19].

While the final USPSTF recommendation does not provide guidance regarding testing for specific clinical conditions, it does state that persons who were born in countries with increased TB prevalence are at increased risk of LTBI and recommends LTBI testing in this population [19]. Our results suggest that many providers are aware of their foreign-born patients’ TB/LTBI risk and are conducting TST/IGRA testing accordingly. We found that as the percentage of foreign-born individuals in a person’s county increased, the likelihood of testing also increased. Similarly, as the state TB rate increased, so did the likelihood of TST/IGRA testing. This corresponds with a greater risk of TB exposure for patients living in high-incidence states and suggests a greater awareness of TB/LTBI among private sector providers practicing in these states.

We found increased likelihood of TST/IGRA testing in pre-kindergarten age (0–4 years) and college-entry age groups (15–19 and 20–24 years) (adjusted probabilities of 9.0%, 12.4% and 8.5%, respectively). These findings align with the practice of requiring that students be screened prior to or upon entry into school [35, 36]. Compared to other age groups, the college-entry years were associated with the highest likelihood of TST/IGRA testing. This is likely because targeted LTBI testing is especially important for colleges and universities. Many foreign-born students are at risk for TB, yet federal regulations do not require people entering the US on student visas to be tested for TB; further, dormitories provide a congregate environment in which TB can be transmitted [36, 37].

While targeted testing of at-risk children and young adults entering or attending school can effectively contribute to domestic TB prevention efforts and is cost-effective, school-related universal LTBI testing is not recommended [38]. While universal testing of this population has been widely conducted [35, 36], there are signs that some local and organizational policies are changing to align with national screening guidelines. For example, Los Angeles County implemented a new testing policy in July 2012, discontinuing universally required pre-kindergarten LTBI testing and beginning risk assessment and targeted testing [39].

Although claims data provide a rich source of information about health conditions and TST/IGRA testing, these data have limitations. We cannot determine when TST/IGRA testing is for employment purposes or if persons tested are employed in high-risk environments. Similarly, while it is evident that TST/IGRA testing in the private sector is occurring at relatively high rates in age groups associated with school entry, we cannot determine whether universal testing or targeted testing was occurring in these groups because we cannot know if pre-testing risk assessments were conducted. Some risk factors are not evident in claims data, including homelessness, visiting areas with high TB prevalence, and residence in congregate settings [21, 25]. Country of birth and household income were also not available through billing data, although we incorporated county-level proxies of these important variables. Further, while we were able to leverage county-level proxies for foreign-birth, we were unable to specifically examine newly hired immigrants from high risk countries. While data limitations disallowed us from examining some risk factors, the current study provides insight into the TST/IGRA testing associated with many important clinical risk factors.

Further, health conditions are only reflected in claims data if they are diagnosed or treated, so undiagnosed and untreated risk factors are not reflected in these analyses. Additionally, claims data do not provide direct assessments of provider knowledge, so our conclusions regarding providers’ awareness of TB risk are based on inference. Our methods do not examine temporality. That is, we do not determine if a TST/IGRA was conducted before or after a diagnosis was assigned or a treatment occurred. We also do not examine the association between risk factors and the receipt of a TST versus an IGRA; we examine the two types of tests in combination.

While CPT codes are generally required for third party payer reimbursement for office-based services, there is not a strong incentive for providers to consistently request reimbursement for TSTs, given the low amount reimbursed for these tests (e.g., the 2012 Medicare Physician Fee Schedule amount for a TST was $7.83) [40]. It appeared that some providers did not include testing CPT codes on all claims in which testing was conducted. Consequently, we inferred some of the TST testing in our analyses using the “special screening examination for pulmonary tuberculosis, including diagnostic skin testing” diagnosis rather than observing the tests in service codes. That we found claims with that diagnosis code and no CPT procedure code accompanying it indicates that TST/IGRA coding is imperfect. It is possible that commercially insured patients are receiving testing that is not documented at all in claims data. Thus, our results may be considered a low estimate of testing activity in the private sector. Our data also excludes testing not submitted to or reimbursed by commercial payers (e.g., testing conducted in workplaces or public health departments). Despite these limitations, commercial claims provide the public health community a window into the TST/IGRA testing occurring in the private sector, and the large sample size enables us to examine low-prevalence risk factors and identify subtle variations in testing practices.

While our study was specifically designed to determine whether TB/LTBI risk factors are associated with an increased likelihood of LTBI testing, our analyses generated additional questions that remain unexplored. We observed that some individuals received >1 TST/IGRA test in the periods studied. It is plausible that patterns of routine testing required for administrative purposes may be evident in the claims data. Similarly, retesting after an initial positive test may also be apparent. Future research exploring retesting patterns and whether these patterns are associated with TB/LTBI risk factors is warranted.

Given changes in local screening requirements [39], the recently released USPSTF recommendations and WHO guidelines [19, 34] and the forthcoming ATS/IDSA/CDC clinical practice guidelines [13], the period we studied reflects screening occurring during a time of shifting clinical practice and policies. This study serves as a baseline measure of LTBI screening in the private sector prior to USPSTF guidelines. The methods used in the current study can be applied to claims data from other time periods and our results can be used to assess whether TST/IGRA testing is increasing or decreasing in high-risk and intermediate-risk groups. Understanding these trends is especially important because there is evidence that the prevalence of LTBI testing in some high-risk groups may be decreasing [41].

## Conclusions

We identified that LTBI testing in the private sector is not uncommon, and that private sector providers appear to have an awareness of TB risk factors. There is a need for clear clinical guidance regarding LTBI testing for US private sector patients with intermediate-risk conditions [18, 19]. Additionally, our analysis indicates that LTBI screening of students before or upon school entry remains common, despite the fact that many students are at low risk of *M*. *tuberculosis* infection. Our findings suggest that continued messaging regarding the importance of targeted school-based testing rather than universal testing is necessary.

Our findings give evidence of the value of the private healthcare sector as a powerful resource in the fight against domestic TB. Private sector healthcare already has extended the reach of public health goals farther than most appreciate. Given the private healthcare system’s broad coverage and massive capacity, there is great potential to further leverage this system towards domestic TB elimination. Our results provide public health leaders and policy-makers in the US with important information about private sector LTBI testing practices, which facilitates the development of programs to shape TB prevention activities in this setting of increasing importance to domestic TB elimination efforts.

## Supporting information

S1 File Codes and logic used to identify TST and IGRA testing. This Excel file contains the specific diagnosis and procedure codes used to identify Tuberculin Skin Testing and Interferon Gamma Release Assay testing.
(XLS)

S2 File Independent variable code lists. This Excel file contains the specific diagnosis and/or procedure codes and logic used to define each independent variable in the multivariable model. Each tab describes a different variable.
(XLS)
